# Severe imported malaria in children in France. A national retrospective study from 1996 to 2005

**DOI:** 10.1371/journal.pone.0180758

**Published:** 2017-07-27

**Authors:** Pierre Mornand, Catherine Verret, Philippe Minodier, Albert Faye, Marc Thellier, Patrick Imbert, the Pediatric Imported Malaria Study Group for the ‘Centre National de Référence du Paludisme’

**Affiliations:** 1 Service de pédiatrie générale, Hôpital d’enfants A. Trousseau, 26 avenue du Dr Arnold Netter, 75571 Paris cedex 12, France; 2 Institut de Recherche Biomédicale des Armées. BP 73, Brétigny Sur Orge Cedex, France; 3 Urgences pédiatriques, CHU Nord, Chemin des Bourrely, Marseille, France; 4 Assistance Publique des Hôpitaux de Paris, Service de Pédiatrie Générale, Hôpital Robert Debré, Paris, France; 5 INSERM 1123, Université Paris Diderot, Paris Sorbonne Cité, Paris, France; 6 AP-HP, Service de Parasitologie-Mycologie, Centre National de Référence du paludisme, Hôpital Pitié-Salpêtrière, Paris, France; 7 INSERM, Centre d’Immunologie et des Maladies Infectieuses de Paris, CIMI-PARIS, U 1135 INSERM/UPMC, Paris, France; 8 Centre de vaccinations internationales, Hôpital d’instruction des armées Bégin, Saint-Mandé, France; George Washington University School of Medicine and Health Sciences, UNITED STATES

## Abstract

**Backgrounds:**

Malaria is a leading cause of imported febrile illnesses in pediatric travelers, but few studies have addressed severe imported pediatric malaria. We aimed to determine the risk factors and the features of imported pediatric severe malaria.

**Methods:**

We conducted a retrospective, descriptive study using the French National Reference Center for Imported Malaria database, in children aged 0–15 years who were hospitalized with a *falciparum* malaria from January 1^st^ 1996 to December 31^th^ 2005.

Uncomplicated and severe cases of *falciparum* malaria were compared to identify risk factors for severe cases. In the hospitals that reported more than five severe cases during the study period, we evaluated severe cases for prognostic factors and assessed the accuracy WHO criteria for predicting severity. Given the rarity of deaths, adverse outcomes were defined as requiring major therapeutic procedures (MTPs)—e.g., sedation, mechanical ventilation, nasal oxygen therapy, blood transfusions, hemodialysis, fluid resuscitation—or pediatric intensive care unit (PICU) admission.

**Results:**

Of 4150 pediatric malaria cases included in the study, 3299 were uncomplicated and 851 (20.5%) were severe. Only one death was recorded during this period. Predictors for severe *falciparum* malaria were: age <2 years (OR = 3.2, 95% CI = 2.5–4.0, p <0.0001) and a travel in the Sahelian region (OR = 1.7, 95% CI = 1.3–2.0, p = 0.0001). Of 422 severe malaria cases, a stay in a Sahelian region, lack of chemoprophylaxis, age <2 years or thrombocytopenia <100 x 10^3/mm^3 predicted adverse outcomes. Except for the hyperparasitemia threshold of 4%, the main WHO 2000 criteria for severe malaria reliably predicted adverse outcomes. In our study, the threshold of parasitemia most predictive of a poor outcome was 8%.

**Conclusion:**

In imported pediatric malaria, children younger than 2 years deserve particular attention. The main WHO 2000 criteria for severity are accurate, except for the threshold of hyperparasitemia, which should be revised.

## Introduction

According to the World Health Organization (WHO), malaria-related mortality has declined significantly for a decade. However in 2014 malaria was responsible for about 438,000 deaths worldwide, mostly children under 5 years of age living in sub-Saharan Africa [1].

Malaria-related deaths are mainly due to *Plasmodium falciparum* infections. The definition of severe malaria was updated by WHO in 2000, revised in 2010 and again in 2015 [2, 3].

Among Western countries, France has the highest incidence of imported malaria both in children and adults [4, 5]. Since the early 2000s, the annual incidence has declined, but the rate of severe cases has not decreased. Management of imported falciparum malaria in France was updated in 2007 [6]. Using data from adults with severe imported malaria, the relevance of the WHO criteria for severe malaria can be assessed in this context and accurate criteria for severity were defined in adult travelers with imported malaria [6, 7]. However, given the scarcity of publications and small size of the pediatric series of severe imported malaria [4, 8, 9], such an assessment in children was not possible. Thus in France, severe pediatric imported *falciparum* malaria has continued to be assessed using the 2000 WHO criteria.

The main objectives of this study were to identify risk factors for severe imported pediatric malaria in France, and to identify predictors of adverse outcomes.

## Methods

### Study sites and population

We conducted a retrospective study using data from the National Reference Centre of Imported Malaria (“CNR du paludisme”), a national network of hospital correspondents who report their cases of imported malaria. The “CNR du paludisme” gives estimations of the annual incidence of imported malaria in mainland France [http://cnrpaludisme-france.org/pages/rapports-annuels/].

All cases reviewed for this study occurred between 1 January 1996 to 31 December 2005 in mainland France in children aged 0–15 years, and were extracted from the national database.

First uncomplicated and severe pediatric malaria cases reported to the “CNR du paludisme” during this period were compared looking for predictors of severe forms.

**Fig 1:**
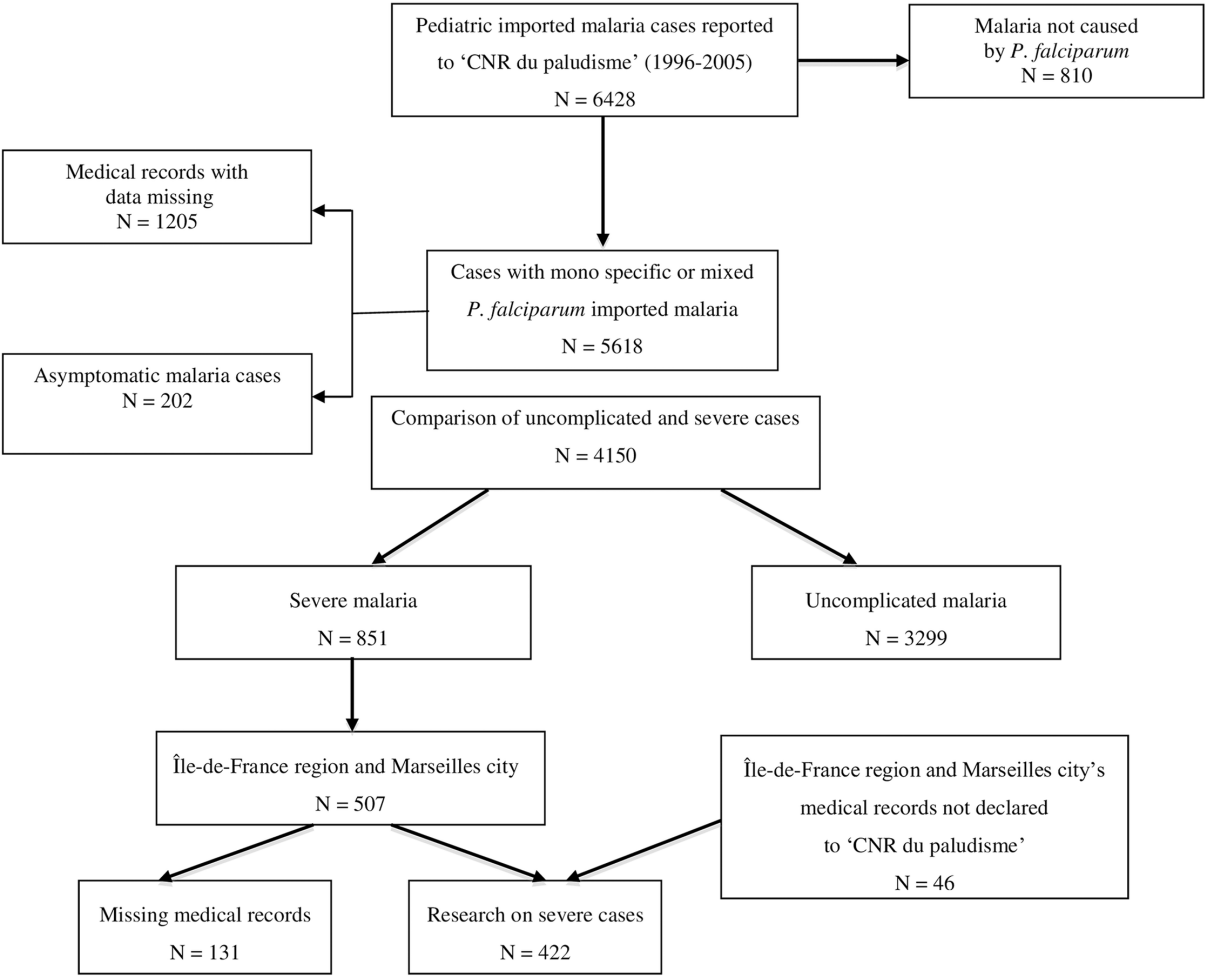
Flow chart of the study: First, comparison between severe and uncomplicated cases registered in the national pediatric database of CNR du paludisme, then research on severe cases in the medical records of hospitals from Region Île-de-France and the city of Marseilles having declared more than 5 severe malaria cases during the period 1996–2005.

Then severe malaria cases (including those with uncomplicated hyperparasitemia) from hospitals that had reported more than 5 severe pediatric malaria cases during the study period were reviewed using a standardized data collection form. In these hospitals, additional cases of severe malaria, not declared to the CNR du paludisme, were found and were added to the cases issued from the national database (Fig 1). Epidemiological, clinical, biological and therapeutic data from the first 24 hours after admission were collected and reviewed to identify predictors of poor outcomes.

### Definitions

The diagnosis of *falciparum* malaria was made by the detection of asexual forms (trophozoites) of *P*. *falciparum*, alone or associated with other *Plasmodium* species, using the examination of thick and /or thin blood smears.

A severe case was defined by the presence of at least one severity criterion according to the 2000 WHO definition, including uncomplicated (i.e., without other WHO 2000 severity criterion) hyperparasitemia ≥ 4%. The 4% threshold was used since almost all reported cases occurred among children born and living in France, and thus considered non-immune [10].

Anemia was defined as haemoglobin (Hgb) <110 g/L and severe anemia as a Hgb <50 g/L or hematocrit <15%. Thrombocytopenia was defined as a blood platelet count <150 x 10^3/mm^3, but we considered a 100 x 10^3/mm^3 threshold for analysis of severity, according to data from non-immune children living in a hypoendemic area [11].

Among severe cases of malaria, multiple organ dysfunction (MOD) were defined by the presence upon hospital admission of at least two of the following organ failure criteria: (i) central nervous system (prostration, disorders of consciousness, convulsions or coma); (ii) respiratory distress; (iii) renal failure, (iv) liver failure or digestive disorders (vomiting, food refusal or abdominal pain); (v) thrombocytopenia <150 x 10^3/mm^3 or spontaneous bleeding.

The countries where *falciparum* malaria was contracted were located in West Africa, Central Africa, Southern and East Africa, and the Indian Ocean (i.e., Madagascar or Comoros Islands). These countries were classified according to epidemiological strata [12]. This classification system is based on transmission dynamics according to Boyd’s classification system [13]. We used 4 main strata: Equatorial (Guinea-Conakry, Ivory Coast, Togo, Benin, Ghana, Nigeria, Tanzania, Rwanda), Tropical (Central African Republic, Cameroon, Gabon, Congo, Zaïre), Sahelian & Sub-desert (Senegal, Mali, Niger, Mauritania, Burkina Faso, Chad, Gambia, Madagascar) and Austral (Comoros Islands and Madagascar).

The adequacy of chemoprophylaxis was assessed according to adherence to French recommendations, depending on the country visited and the season of exposure, which are published every year under the authority of the High Council of Public Health (*Haut Conseil de Santé Publique*). During the period of the study, recommendations were adapted to the level of sensitivity of antimalarial drugs: zone 0 (no malaria); zone 1 (no chloroquine resistance): chloroquine; zone 2 (prevalence of chloroquine resistant malaria): proguanil or atovaquone/proguanil; zone 3 (high prevalence of chloroquine- or multi-resistant malaria): atovaquone/proguanil or mefloquine or doxycycline.

The French recommendations for the treatment of children malaria during the period of study were halofantrine or mefloquine in uncomplicated malaria or quinine intravenously without loading dose in severe malaria (http://www.infectiologie.com/UserFiles/File/medias/_documents/consensus/palu99.pdf).

Given the rarity of deaths in our series, a “poor outcome” was defined as (i) the need for at least one major therapeutic procedure (MTP) including sedation, mechanical ventilation, nasal oxygen therapy, blood transfusions, hemodialysis or fluid resuscitation, or (ii) an admission to a pediatric intensive care unit (PICU).

### Statistical methods

Statistical analyses were performed using StataTM version 9 software.

The comparison of variables required the use of a chi 2 test or Fisher exact test under the conditions of applications. The mean comparison was made from the Student's t tests.

Variables included in the univariate and multivariate analyses were age, sex, geographical distribution, chemoprophylaxis and platelet count.

The search for predictors of poor outcomes, defined by a requirement for a major therapeutic procedure or PICU admission, was performed using a logistic regression model. A first step was to select the variables (age, gender, countries visited, chemoprophylaxis and platelet count) associated with the endpoint (in severe forms the use of MTP, PICU admission) in univariate analysis. Factors associated with a *p*-value < 0,25 were selected for multivariate analysis. The variables were selected by a backward stepwise method (Hosmer and Lemeshow). Only significant variables at 5% were retained at the end of this analysis.

The criteria for severe malaria [2] were more intensively studied. For each criterion, the association with the endpoint was evaluated by univariate model and adjusted on the previously determined predictive model.

Parasitemia was evaluated using several definitions: dichotomous criterion (parasitemia ≥4% *versus* <4%), variable in three classes (<4%; 4–9%; ≥10%) and continuous variable. Regarding the analysis of parasitemia as a continuous variable, a ROC curve (Receiver Operating Characteristic Curve) was drawn (representation of the sensitivity *versus* 1 minus the specificity) to determine the most accurate threshold associated with a poor outcome.

### Ethics statement

The ‘CNR du paludisme’ online declaration system for the notification of malaria cases was approved by the National Commission for Informatics and Personal Liberties (‘Commission Nationale de l’Informatique et des Libertés’ or CNIL).

As CNR’s data were anonymous, we could not obtain an informed consent from children or parents. Data collected in hospital files were also given anonymously, so we could not reach children or parents to obtain an informed consent. Finally, the study was designed in 2004–2005. In 2004, August, French law introduced the ‘Comité consultatif sur le traitement de l'information en matière de recherche dans le domaine de la santé’, as a national ethics committee for observational studies. That committee was not fully operational when we conducted this study. All the data were collected retrospectively and anonymized in a standardized case report form in the database.

## Results

### Characteristics of the overall study population

From 1996 to 2005, 4,150 cases of pediatric malaria were reported to the CNR du paludisme, of which 3,299 were uncomplicated and 851 were severe (Fig 1).

Reports of imported malaria have tended to decrease since 1999. However, the annual ratio of severe to uncomplicated forms remained stable during the study period.

The distribution of the epidemiological and biological characteristics of the patients is shown in Table 1.

**Table 1:** Distribution of main characteristics of children with uncomplicated or severe forms of imported malaria in mainland France, 1996–2005.

Characteristics	Uncomplicated form	Severe form	*p*
	(N = 3299)	(N = 851)	
**Epidemiology**			
Sex ratio (M/F)	1.2	1.1	NA^*^
Mean age (years)	7.7 ± 4.2	6.0 ± 4.1	< 0.0001
Resident in France (%)	77	82	< 0.002
Epidemiological strata (%)			
*Tropical*	37.2	35.5	NA^*^
*Sahelian and Sub-desert*	25.1	29.8	NA^*^
*Equatorial*	22.6	17.9	NA^*^
*Austral* **	15.1	16.8	NA^*^
**Correct chemoprophylaxis**	6.0	5.8	NA^*^
**Delay in diagnosis (days)**	6.2 ± 10.5	5.0 ± 14.5	0.007
**Biology**			
Hgb rate (g/L)	102 ± 20	97 ± 23	0.0001
Parasitemia (%)	1.0 ± 1.0	8.2 ± 6.8	0.0001
Platelet count (x10^3/mm^3)	1.0 ± 1.0	8.2 ± 6.8	0.0001

*: Not applicable

**: Comoros Islands and Madagascar

Only 14 children (0.4%) had visited non-African regions (Asia = 8; Caribbean = 3, Latin America = 2; Pacific = 1), none with severe malaria. During their stay, 36% of children received no chemoprophylaxis. Only 6% of parents regularly gave their children prophylaxis consistent with French national recommendations (updated for resistance.

Oral antimalarial treatments were halofantrine (70.7%), quinine (16.1%) or mefloquine (10.6%). Halofantrine was used most commonly but usage declined steadily from 1996 to 2005: 85% to 47% in uncomplicated cases, and 67% to 28% in severe cases, respectively. Intravenous quinine was used more often in severe cases (26.6%) than in uncomplicated forms (13.4%) (p = 0.0001) over the study period. Mefloquine was increasingly prescribed for both forms from 1996 to 2005: 3% to 25% in uncomplicated cases, and 2% to 33% in severe cases, respectively.

All patients with uncomplicated malaria recovered uneventfully. Among the severe cases, 838 children (98.5%) had an uneventful course, 11 patients (1.3%) had transient clinical worsening, one (0.1%) had neurological sequels and one (0.1%) died.

### Factors associated with the occurrence of severe malaria

Comparison between uncomplicated and severe malaria was performed in univariate analysis. The proportion of severe cases was significantly higher among children travelling in a Sahelian country than in those returning from other countries (29.8% *versus* 25.1%, p = 0.002) and among children under two years (21% *versus* 11%, p = 0.0001).

The rates of chemoprophylaxis compliance were comparable between uncomplicated and severe malaria.

The average parasitemia was significantly lower in uncomplicated cases than in severe cases (1.0 ± 1.0% *versus* 8.2 ± 6.8%, p = 0.0001). The mean rate of Hgb was significantly lower in severe than in uncomplicated malaria (97 ± 23 g/L *versus* 102 ± 20 g/L, p = 0.0001). The mean platelet count was also significantly lower in severe than in uncomplicated cases (126 ± 89 x 10^3/mm^3 *versus* 176 ± 102 x 10^3/mm^3, p = 0.0001).

**Fig 2:**
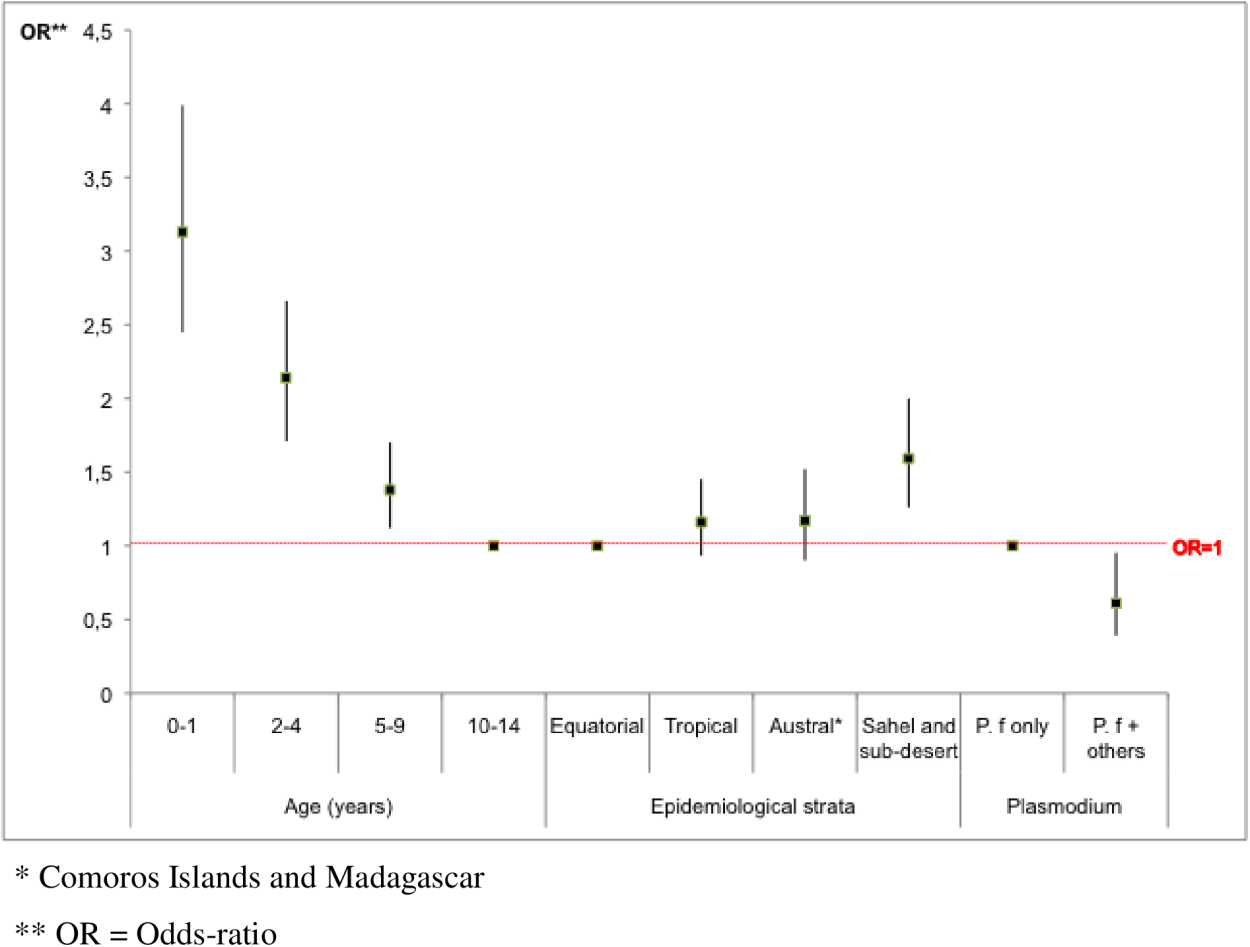
Predictors for the occurrence of severe cases in 4150 children hospitalized with malaria in mainland France, 1996–2005.

In multivariate analysis, independent predictors for the occurrence of severe malaria were age <2 y (OR = 3, p<0.0001) and a stay in Sahel (OR = 2, p = 0.0001). Conversely, mixed *Plasmodium* species infections were associated with a lower risk of severe malaria, compared to *falciparum* monospecific-infections (OR = 2, p = 0.02) (Fig 2).

### Description of the children with severe malaria

During the study period, 14 hospitals reported at least five cases of severe pediatric malaria, including 12 in the Region Île-de-France and 2 in the city of Marseilles, yielding 422 cases that were included in a detailed analysis of severe malaria cases (Fig 1).

The annual incidence has generally decreased since 1999. The average patient age was 5.8 ± 4 years and the sex ratio (M/F) was 0.9. In our series, most of children (90.7%) lived in France. The most visited countries were the Ivory Coast (22%), the Comoros Islands (20%), Mali (16%), Senegal (13%) and Cameroon (9%). The average length of stay was 64 ± 97 days.

Globally, 73% patients were reported (by themselves or parents) as taking chemoprophylaxis during their stay. Of these, 44 children (10.4%) were fully compliant with recommendations. The use of vector control measures (bed nets, skin repellents) was commented on in 159 children, of whom 16% were reported sleeping under a bed net and 10% using repellents.

The average time from the onset of symptoms to diagnosis malaria was significantly longer in uncomplicated than in severe malaria cases (6.2 ± 10.5 days versus 5.0 ± 14.5 days, p = 0.007).

Besides WHO criteria, clinical signs present on admission were: temperature ≥38.5°C (64%), hepatomegaly (26%), vomiting (25%), splenomegaly (23%) and headache (19%). Only 2 patients had pupillary abnormalities (bilateral myosis or unreactive pupils). Co-infection was diagnosed in 31 children: gastrointestinal infections (*Salmonella sp*., *Campylobacter jejuni*, *Shigella sp*.) (n = 12), hepatitis A (n = 8), urinary tract infection (n = 4), ringworm (n = 4) and viral infection (EBV, HSV) (n = 3). No case of bacteremia was noted.

**Fig 3:**
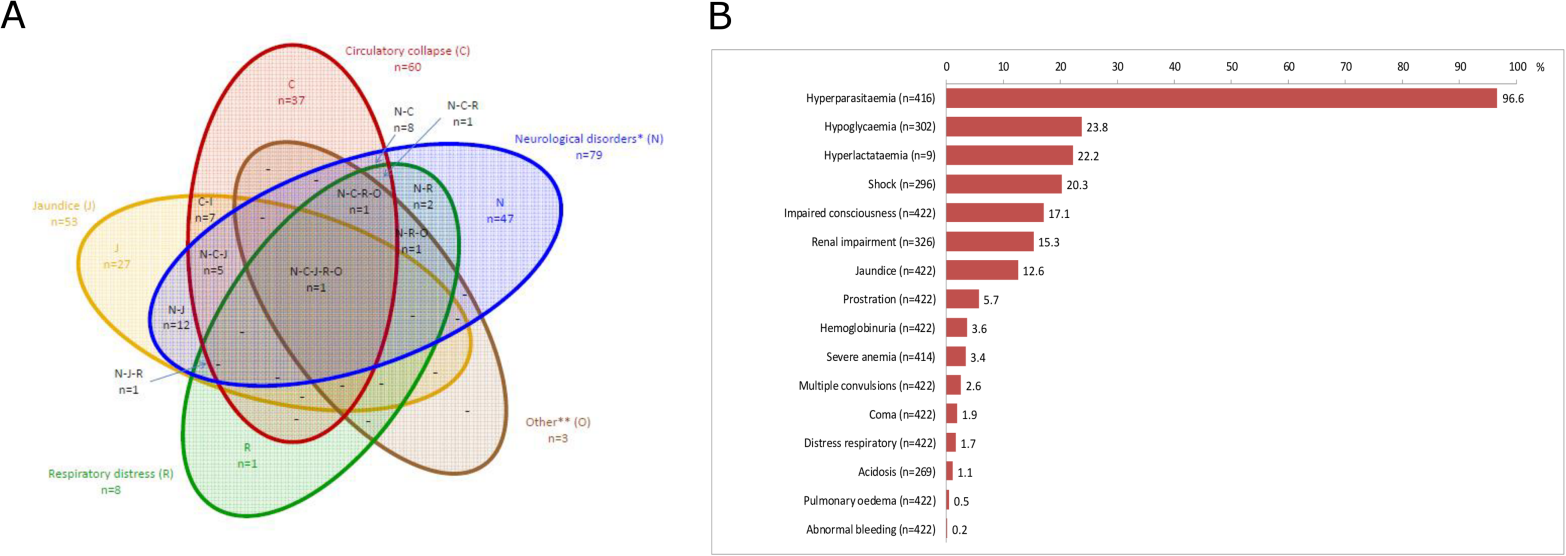
Distribution of the 2000 WHO criteria for severity in 422 children admitted for severe imported malaria in mainland France, 1996–2005 (Fig 3A). Prevalence and overlap of the WHO clinical criteria at admission in 422 children with severe imported malaria in mainland France, 1996–2005 (Fig 3B).

The WHO clinical criteria for severity analyzed in this series are shown in Fig 3A and 3B. The median parasitemia was 6% (interquartile [IQ] 25–75: 5–10; range: 0.01–50). Parasitemia was ≥4% in 404 children (97%) and uncomplicated in 219 (52%). Hgb was <110 g/L in 301 (73%) patients, and <50 g/L in 13 (3.1%). Only two children among the 269 tested had acidosis (range of bicarbonate level: 13–29 mmoL/L).

Children had one to six clinical criteria of severity at admission (Fig 3B). At least 2 clinical criteria of severe malaria cases were seen in 159 cases (58,6%). Most frequent clinical association were neurological disorders and jaundice (N = 12).

Platelet count was <150 x 10^3/mm^3 in 283 (68%) children, and <100 x 10^3/mm^3 in 185 (45%).

Treatments were halofantrine (n = 302 patients, 72%, of whom 142 [47%] received a second course 14 days later), intravenous quinine (n = 81 patients, 19%, of whom 5 [6.3%] received a loading dose), or mefloquine (n = 40 patients, 9.5%). No severe side effects were recorded.

Of 422 patients, 100 (23.7%) required at least one MTP (blood transfusion 16.6%, volume expansion 12.3%, oxygen 5%, sedation 3%, mechanical ventilation 2.6%) and 43 (10%) stayed in a PICU. The distribution of numbers of units of transfusions received across all subjects was: 1 unit (N = 51), 2 units (N = 13), 3 units (N = 3), > 3 units (N = 2). Two children received massive red blood cell transfusions, i.e., 15 units each.

The clinical course was uneventful in 366 children (86.7%). Thirty children (8.2%) had a relapse within 28 days of the first episode, usually after a single course of halofantrine (60% of relapses). All relapses were uncomplicated and patients fully recovered using oral antimalarial drugs. Finally, only one child died and another had neurological sequelae (language disorders and epilepsy persisting after a 2-year follow-up).

The child who died presented at admission with impaired consciousness, renal failure, pulmonary oedema, and thrombocytopenia (25 x 10^3/mm^3). The child with neurological sequelae presented with hyperparasitemia, impaired consciousness and hypoglycemia.

#### Factors associated with an adverse outcome in children with severe malaria

In a univariate analysis, among 422 children, 54 cases were not included due to missing data.

275 children had no need for MTP, while 93 required at least one MTP. The sex ratio (M/F) was comparable regardless of whether or not MTP was used (1.1 *versus* 0.9, respectively). The average age tended to be higher in children with MTP than in those without MTP (5 ± 4 years *versus* 6 ± 4 years, p = 0.06).

40 children were admitted to a PICU while 328 children were not. The latter were more likely to be male than the others (sex ratio M/F: 1.4 *versus* 0.9, p = 0.16). The average age was comparable in both groups (6.3 ± 4.6 years *versus* 5.7 ± 4.0 years, p = 0.36).

In multivariate analysis, a stay in a Sahelian country, lack of chemoprophylaxis, and a thrombocytopenia <100 x 10^3/mm^3 were independent predictors both for the use of MTP and ICU admission. Young age (<2 years) was associated with the use of MTPs but not admission to an ICU (Tables 2 and 3).

**Table 2:** Univariate and Multivariable analysis: Predictive factors for the use of major therapeutic procedures in 368 children admitted for severe imported malaria in mainland France, 1996–2005.

*Variables*	No use of MTP*	Use of MTP	Univariate analysis				Multivariable analysis		
	(n = 275)	(n = 93)							
	N (%)	N (%)	OR	IC-95%		*P*	Adjusted OR	IC-95%	*P*
***Age (years)***						0.12			0.02
0–1	51 (18.6)	28 (30.1)	2.1		1.0–4.2		3.3	1.5–7.5	
2–4	85 (30.9)	23 (24.7)	1.0				1.4	0.6–3.1	
5–9	84 (30.6)	27 (29.0)	1.2		0.6–2.4		1.5	0.7–3.3	
10–14	55 (20.0)	15 (16.1)	1.0				1.0		
***Epidemiological strata***						0.02			0.003
Sahelian	82 (29.8)	41 (44.1)	1.5		0.7–3.3		2.1	0.9–4.6	
and Sub-desert									
Tropical	101 (36.7)	19 (20.4)	0.6		0.3–1.3		0.6	0.3–1.4	
Equatorial	37 (13.5)	12 (12.9)	1.0				1.0		
Austral**	55 (20.0)	21 (22.6)	1.2		0.5–2.7		1.1	0.4–2.5	
***Chemoprophylaxis***						0.16			0.05
Adapted and regular	33 (12.0)	11 (11.8)	1.0				1.0		
Inadequate or irregular	175 (63.6)	50 (53.8)	0.9		0.4–1.8		0.8	0.4–1.9	
No treatment	67 (24.4)	32 (34.4)	1.4		0.6–3.2		1.7	0.7–4.0	
***Platelet count (***x 10^3/mm^3)						0.07			0.03
≥ 150	89 (32.4)	20 (21.5)	1				1.0		
100–150	71 (25.8)	22 (23.7)	1.4		0.7–2.7		1.3	0.6–2.5	
< 100	115 (41.8)	51 (54.8)	2.0		1.1–3.5		2.1	1.1–4.0	

*: MTP = major therapeutic procedures (including sedation, mechanical ventilation, nasal oxygen therapy, blood transfusions, hemodialysis or fluid resuscitation)

**: Comoros Islands and Madagascar

**Table 3:** Univariate and Multivariablee analysis: Predictive factors for PICU stay in 368 children admitted for severe imported malaria in mainland France, 1996–2005.

*Variables*	No PICU* stay (n = 328)	PICU stay	Univariate analysis			Multivariable analysis		
		(n = 40)						
	N (%)	N (%)	OR	IC-95%	*P*	Adjusted OR	IC-95%	*P*
***Sex***					0.21			0.07
Male	154 (47.0)	23 (57.5)	1.0			1.0		
Female	174 (53.0)	17 (42.5)	0.7	0.3–1.3		0.5	0.3–1.0	
***Epidemiological strata***					0.18			0.03
Sahelian	104 (31.7)	19 (47.5)	4.3	1.0–19.2		6.6	1.4–31.7	
and Sub-desert								
Tropical	108 (32.9)	12 (30.0)	2.6	0.6–12.1		3.0	0.6–14.5	
Equatorial	47 (14.3)	2 (5.0)	1.0			1.0		
Austral**	69 (21.0)	7 (17.5)	2.4	0.5–12.0		2.6	0.5–13.6	
***Chemoprophylaxis***					0.06			0.04
Adapted and regular	41 (12.5)	3 (7.5)	1.0			1.0		
Inadequate or irregular	205 (62.5)	20 (50.0)	1.3	0.4–4.7		1.2	0.3–4.4	
No treatment	82 (25.0)	17 (43.6)	2.8	0.8–10.2		3.0	0.8–11.4	
***Platelet count (***x 10^3/mm^3)					0.002			0.001
≥ 150	103 (31.4)	6 (15.0)	1.0			1.0		
100–150	88 (26.8)	5 (12.5)	1.0	0.3–3.3		0.9	0.3–3.2	
< 100	137 (41.8)	29 (72.5)	3.6	1.4–9.1		4.0	1.6–10.4	

*: PICU = admission to a pediatric intensive care unit

**: Comoros Islands and Madagascar

#### Prognostic relevance of 2000 WHO severity criteria

WHO criteria present on admission or within the first 24h after admission were evaluated by univariate and multivariate analysis.

Regarding the use of MTPs and PICU admission, sensitivity (Se), specificity (Spe), positive predictive value (PPV) and highest negative predictive value (NPV) are shown in Tables 4 and 5.

**Table 4:** Sensitivity (Se), specificity (Spe), positive (PPV) and negative (NPV) predictive values of the WHO 2000 severity criteria for the use of Major Therapeutic Acts (Table 4A) in 422 children admitted for severe malaria in metropolitan France between 1996 and 2005.

WHO criteria	N	No use of MTP*	Use of MTP	Sensitivity	Specificity	NPV**	PPV***
Coma	422	0.3%	7.0%	7.0%	99.7%	87.5%	77.5%
Impaired consciousness	422	8.7%	44.0%	44.0%	91.3%	61.1%	84.0%
Convulsions	422	0.9%	8.0%	8.0%	99.1%	72.7%	77.6%
Prostration	422	1.9%	18.0%	18.0%	98.1%	75.0%	79.4%
Respiratory distress	422	0.0%	8.0%	8.0%	100.0%	100.0%	77.8%
Hypoglycemia	302	20.1%	35.6%	35.6%	79.9%	36.1%	79.6%
Jaundice	422	9.9%	21.0%	21.0%	90.1%	39.6%	78.6%
Metabolic acidosis	269	0.5%	2.8%	2.8%	99.5%	66.7%	74.1%
Hyperlactatemia	9	-	22.2%	22.2%	-	100.0%	0.0%
Severe anemia	414	0.3%	13.1%	13.1%	99.7%	92.9%	78.5%
Hemoglobinuria	422	2.2%	8.0%	8.0%	97.8%	53.3%	77.4%
Renal failure	326	13.7%	20.0%	20.0%	86.3%	34.0%	75.4%
Circulatory collapse	296	0.0%	3.8%	3.8%	100.0%	100.0%	73.7%
Spontaneous bleeding	422	0.0%	1.0%	1.0%	100.0%	100.0%	76.5%
Pulmonary edema	422	0.0%	2.0%	2.0%	100.0%	100.0%	76.7%
Hyperparasitemia >4%	416	97.8%	92.6%	92.6%	2.2%	21.9%	50.0%

*: MTP = major therapeutic procedures (including sedation, mechanical ventilation, nasal oxygen therapy, blood transfusions, hemodialysis or fluid resuscitation)

**: NPV = negative predictive value

***: PPV = positive predictive value

**Table 5:** Sensitivity (Se), specificity (Spe), positive (PPV) and negative (NPV) predictive values of the WHO 2000 severity criteria for the admission in PICU in 422 children admitted for severe malaria in metropolitan France between 1996 and 2005.

WHO criteria	N	No PICU*	PICU	Sensitivity	Specificity	NPV**	PPV***
Coma	422	0.5%	14.0%	14.0%	99.5%	75.0%	91.1%
Impaired consciousness	422	11.6%	65.1%	65.1%	88.4%	38.9%	95.7%
Convulsions	422	1.3%	14.0%	14.0%	98.7%	54.5%	91.0%
Prostration	422	2.9%	30.2%	30.2%	97.1%	54.2%	92.5%
Respiratory distress	422	0.3%	16.3%	16.3%	99.7%	87.5%	91.3%
Hypoglycemia	302	21.0%	50.0%	50.0%	79.0%	20.8%	93.5%
Jaundice	422	10.8%	27.9%	27.9%	89.2%	22.6%	91.6%
Metabolic acidosis	269	0.4%	8.0%	8.0%	99.6%	66.7%	91.4%
Hyperlactatemia	9	0.0%	25.0%	25.0%	100.0%	100.0%	14.3%
Severe anemia	414	2.4%	11.9%	11.9%	97.6%	35.7%	90.8%
Hemoglobinuria	422	3.2%	7.0%	7.0%	96.8%	20.0%	90.2%
Renal failure	326	13.7%	29.4%	29.4%	86.3%	20.0%	91.3%
Circulatory collapse	296	0.0%	8.8%	8.8%	100.0%	100.0%	89.4%
Spontaneous bleeding	422	0.0%	2.3%	2.3%	100.0%	100.0%	90.0%
Pulmonary edema	422	0.0%	7.0%	7.0%	100.0%	100.0%	90.5%
Hyperparasitemia >4%	416	97.9%	85.0%	85.0%	2.1%	8.5%	57.1%

*: PICU = admission to a pediatric intensive care unit

**: NPV = negative predictive value

***: PPV = positive predictive value

**Fig 4:**
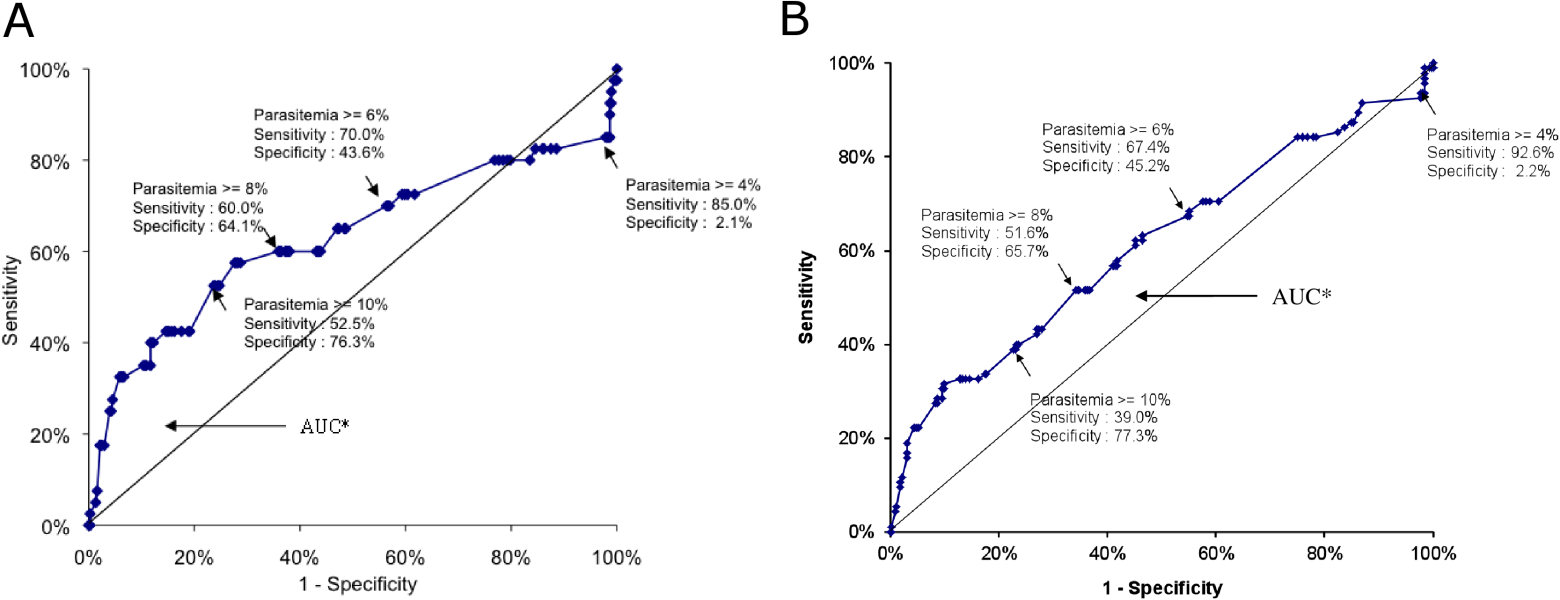
ROC curves: Assessment of a parasitemia threshold for the prediction of the use of major therapeutic procedures (Fig a), or PICU stay (Fig b) in 368 children admitted for severe imported malaria in mainland France, 1996–2005.

Hyperparasitemia, at the 4% threshold, was not an independent risk factor for the use of MTPs (OR: 0.3; 95% CI: 0.1 to 1.1, p = 0.07) or PICU admission (OR: 0.3; 95% CI: 0.1 to 1.1, p = 0.06). We tried to determine the most accurate threshold of parasitemia for predicting severity, defined as the use of MTPs and / or PICU admission. According to the ROC curves (Fig 4), a 4% parasitemia was not discriminatory. However the 8% threshold (for the use of MTP: Se: 51.6%; Spe: 65.7%; AUC: 0.613 [CI 95%: 0.543–0.682], and PICU admission: Se: 60%; Spe: 64.1%; AUC: 0.629 [CI 95%: 0.514–0.744]) was the most accurate for predicting severity in our series.

## Discussion

Very few data are available on severe pediatric imported malaria. To our knowledge, this study is the largest ever reported.

The annual incidence of severe pediatric malaria in France during the period of our study followed the trends observed in Western countries. After a steady increase during the ‘90s, attributed to the increase in air traffic to endemic areas, a gradual decline in incidence was seen after 2000 despite an increasing number of travelers to the areas at risk [5]. This paradox could be partly explained by increased adherence to malaria prevention strategies in the past few years [14], compared to previous pediatric data from France or other European countries [4, 6, 15]. Another possibility is the significant reduction in the incidence of malaria in the past 10 years in many African countries and the resultant reduction in risk for the travelers [1].

Of note we observed practitioner’s difficulties in identifying severe malaria, leading to inappropriate therapeutic strategies such as administering antimalarial drugs orally for the treatment of severe cases. This emphasizes the importance of identifying risk factors for severe malaria in pediatric travelers as well as specific prognostic factors in severe cases.

The overall lethality was 0.1 per 1000 for pediatric cases reported to the CNR du paludisme between 1996 and 2005. In severe cases reported in targeted hospitals, the case fatality rate was 0.2% (1 death / 422 cases). These results are consistent with the lethality of imported malaria, 0.2% according to data reported from 11 Western countries including France, a rate 10 times lower than in adults with imported malaria [5]. Conversely, children living in endemic regions, especially the youngest, suffer a far higher fatality rate [1, 2]. This discrepancy has several possible explanations, including reliable access to the French National Health System, the good general condition of traveling children and an immunological response in children different from that in adults [16].

We compared uncomplicated and severe malaria cases to assess risk factors for severe malaria. Several risk factors were positively associated with severe cases: age <2 years, a stay in the Sahel, and thrombocytopenia. Conversely, mixed *Plasmodium* species infections were negatively associated with severe cases.

In endemic countries, it is well established that young children are at high risk for life-threatening malaria [2].

Why travel in the Sahel is a marker of severity is not readily explained. Host or environmental factors might be involved. Further research is needed, including anthropological studies on the perception of malaria risk and adherence to prevention among travelers, of whom most are “visiting friends and relatives” [4, 5, 17].

Thrombocytopenia is a common finding in non-immune children with malaria, whether living in or returning from endemic areas [6, 11, 18].

In our study, a thrombocytopenia <150 x 10^3/mm^3 was significantly more frequent in severe than in uncomplicated cases of malaria. This finding is consistent with pediatric malaria data both in endemic areas [11, 18–20], as well as with imported malaria [8]. The same finding was reported in adult travelers [6, 10, 21].

Of note, we found that mixed *Plasmodium* species infections were associated with a halving of the risk of severe malaria, compared to monospecific *falciparum* infections (OR, 0.6; 95% CI: 0.4–0.9, p = 0.02). This has been previously reported both in people living in endemic areas as well as in travelers [22, 23]. It has been hypothesized that chronic infection with *P*. *vivax*, a species less life-threatening that *P*. *falciparum*, could reduce the severity of *falciparum* malaria attacks through cross-species immunity. Further studies are needed to confirm this finding, since deaths can occur with mixed *P*. *falciparum*—*P*. *vivax* infections despite the lack of cytoadherence by *P*. *vivax* in brain capillaries [24].

Delayed diagnosis (time from the onset of fever or any symptoms to diagnosis of malaria) remains a concern in imported pediatric malaria. The average delay reported in previous French studies varied from 4.5 to 9.5 days [6]. In our study, children with a severe form of malaria (including uncomplicated hyperparasitemia) sought care earlier than others, as previously described in French children with imported malaria (2 days in severe versus 3 days in uncomplicated malaria).

In endemic areas, delay in seeking care was shown to vary according to the criterion of severity (e.g., earlier in cases of coma than for severe anemia) [25]. This could explain why child travelers in our series suffered no consequences from delayed diagnosis since major criteria of severe malaria, e.g., cerebral malaria or respiratory distress, were rare. Regardless, it is critical to immediately consider malaria in any febrile patients returning from an endemic country [6].

Hyperparasitemia was the most frequent criteria at admission in our study. It was associated especially with neurological disorders or hypoglycemia.

The most frequent association of clinical criteria met in our series was neurological disorders and jaundice (N = 7).

In this study we assessed the relevance of several criteria of severe malaria updated by the WHO in 2000 in predicting severity. Neurologic disorders, i.e., impaired consciousness, coma and convulsions, were the most reliable prognostic indicators in our series, using either the need for MTPs or PICU admission. Coma is the criterion whose reliability is one of the most consistently reported in the pediatric literature [26, 27]. It is associated with a significant risk for death or sequelae both in hypo- and hyper endemic areas [28–30], and was present in the sole fatality in our series. Less severe neurologic manifestations, e.g. impaired consciousness and convulsions are also associated with a poor prognosis in endemic areas [31].

Severe anemia is the most common manifestation of severe malaria in children, whatever the transmission level, and is responsible for the highest number of malarial-related deaths in malarial settings [2, 22, 32]. In our series it was predictive for MTP use (PPV: 93%), but was not associated with deaths, most likely because of the rapid access to blood transfusion. In endemic areas, severe anemia-related deaths are rare in hospitals, due to transfusion availability [18, 25, 26, 30–32]. Conversely, in rural areas where access to transfusions is limited, severe anemia is the leading cause of malaria-related deaths in children [25, 26, 30, 31, 33]. Over 15% of children in our study received transfusions during their hospitalization; all had a Hgb <70 g/L, a threshold French practitioners consider for transfusions, as does WHO for hypoendemic areas [3]. In England, a rate of Hgb <80 g/L is considered a criterion of severity, requiring transfusion [34, 35]. We propose considering malaria-related anemia in a child traveler as severe if the Hgb is <70 g/L.

In our series, respiratory distress was strongly predictive for PICU admission (PPV: 85.7%). This major criterion of severity in children should not be confused with the acute respiratory distress syndrome in adults (ARDS). It is of high prognostic relevance in the pediatric literature from tropical countries, whatever the level of malarial transmission [4, 36].

Acidosis is also predictive of death, especially in endemic zones when combined with either cerebral malaria or severe anemia [37, 38]. In our population, acidosis was not associated with an increased risk of adverse outcomes, possibly due to the lack of power of analysis. The 2007 French guidelines emphasized its strong prognostic value [6], assessed first in children living in endemic areas and recently in adult travelers [21, 39]. This highlights the need for further studies of acidosis in malaria cases with organ dysfunction in pediatric travelers.

In endemic areas, hyperparasitemia was found to be an independent predictor for cerebral malaria and death, especially at ages below 5 years [2, 22, 30, 40]. In a series from Thailand, four children died with a parasitemia> 4% on admission as the only criterion for severity [22]. Recently, Indian authors have found that a parasitemia >10% was predictive for death in children admitted to an ICU [30]. According to the last WHO guidelines, hyperparasitemia is considered a criterion of severity in all the areas of malaria transmission if > 10% [3]. In our series, hyperparasitemia ≥4% was present in 97% of children with a severe form and was the sole WHO criteria in 52% of severe cases. The prognostic value of hyperparasitemia and its effect on clinical management has not yet been assessed in imported pediatric *falciparum* malaria. In the UK, a parasitemia > 2% as a unique criterion of severity is considered to require close monitoring of the child in an ICU [35]. In our series, uncomplicated hyperparasitemia ≥4% was not associated with severity. Moreover, most of the children (75%) with parasitemia ≥4% as the only criterion of severity were cured with oral antimalarial treatment other than Artemisinin based-combination therapies (ACT) (data not shown), without MTPs or PICU admission. According to our study, a level of 8% is much more accurate in predicting severity. This is also more consistent with the 2007 French consensus guidelines which recommend that children with a parasitemia between 4 and 10% as the sole criterion of severity should be kept in emergency unit or pediatrics service and treated with oral antimalarial drugs. ACTs are the best choice in this case, given their action (young trophozoites) very early in the erythrocytic life cycle of the parasite and their very rapid action on the parasite load [3, 22]. Children with uncomplicated hyperparasitemia above 10% should be promptly admitted to a PICU and given intravenous antimalarial therapy, artesunate being the first-line antimalarial drug recommended since 2013 in France [6, 41].

Multivariate analysis of severe cases identified other factors which can help practitioners to assess severity: (i) age <2 y: these children had almost 4 times the risk for MTP use than children ≥2 y, but had the same risk for PICU admission compared to older children. This discrepancy may be explained by the higher frequency of some complications occurring below 2 y, i.e., hypovolemia or severe anemia, which can be corrected with procedures not requiring a PICU admission; (ii) a stay in Sahel: this original finding merits further research; (iii) the lack of chemoprophylaxis: this result is consistent with data from adult travelers [10, 42]; (iv) thrombocytopenia <100 x 10^3/mm^3: this finding is consistent with studies from adult travelers and children or adults living in endemic malarial areas [11, 20, 21, 43–45].

In our series, 159 (37.5%) children admitted for severe malaria met the definition of MOD. These children were more frequently admitted to intensive care because they often required several MTPs, e.g., oxygen therapy and transfusions (data not shown).

No standardized prognostic score has been validated regarding the need for intensive care in imported pediatric malaria, in contrast to the simplified severity index (SAPS II) in adults [7]. In endemic areas, several authors have used various scores in children: PRISM [46], a scoring system too complex for current practice, simplified scores such as Multiple Organ Dysfunction Score (MODS) [36, 47, 48], or the Lambaréné Organ Dysfunction Score (LODS), a score based solely on clinical criteria that has recently been found to be quite useful in Uganda [49]. Further studies in pediatric travelers are needed to identify scoring systems for better evaluation of malaria severity.

Our study has several limitations: (i) Reporting of imported malaria to CNR du paludisme by the members of this network is voluntary. Thus the cases extracted from the national database for our study may not be entirely representative of the total French experience (ii) Our study is retrospective, with missing data; (iii) The study involved cases from 1996–2005, thus including data reflecting now-outdated practices; (iv) The design of our study, i.e., selected hospitals declaring more than 5 severe pediatric malaria cases throughout the period, missed 55.8% of pediatric severe cases registered by the CNR du paludisme, and; (v) we did not assess all the WHO severity criteria as prognostic factors in our study, given their low frequency in our series and/or missing data.

However, we do not think these limitations alter the accuracy of the results. Moreover this study is nationwide over ten years, and is the largest series of imported pediatric malaria to date.

## Conclusion

Among children with malaria after a stay in endemic zones, those at risk for severe malaria are likely to be infants, had visited a Sahelian country, missed chemoprophylaxis, or to present with thrombocytopenia <100 x 10^3/mm^3. Conversely, mixed *Plasmodium* species infection are protective against severe malaria. Among severe cases, a stay in Sahel, absence of chemoprophylaxis, age <2 y, presence of the major 2000 WHO criteria of severity, parasitemia 8% or a thrombocytopenia <100 x 10^3/mm^3 were relevant prognostic factors. This study provides important information to help practitioners in the evaluation and management of severe imported pediatric malaria in France. However these findings should be confirmed by prospective studies.

## Abbreviations

WHO: World Health Organization
MTP: major therapeutic procedures
ACT: Artemisinin-based Combination Therapy
Hgb: haemoglobin
MOD: multiple organ dysfunction
PICU: pediatric intensive care unit
